# Characteristics of maturity onset diabetes of the young in a large diabetes center

**DOI:** 10.1111/pedi.12289

**Published:** 2015-06-08

**Authors:** Christina Chambers, Alexandra Fouts, Fran Dong, Kevin Colclough, Zhenyuan Wang, Sat Dev Batish, Malgorzata Jaremko, Sian Ellard, Andrew T Hattersley, Georgeanna Klingensmith, Andrea K Steck

**Affiliations:** ^1^ Barbara Davis Center for Childhood DiabetesUniversity of Colorado DenverAuroraCOUSA; ^2^ Department of Genetics, Institute of Biomedical and Clinical ScienceUniversity of Exeter Medical SchoolExeterUK; ^3^ Athena DiagnosticsWorcesterMAUSA

**Keywords:** GCK, HNF1A, HNF4A, MODY

## Abstract

Maturity onset diabetes of the young (MODY) is a monogenic form of diabetes caused by a mutation in a single gene, often not requiring insulin. The aim of this study was to estimate the frequency and clinical characteristics of MODY at the Barbara Davis Center. A total of 97 subjects with diabetes onset before age 25, a random C‐peptide ≥0.1 ng/mL, and negative for all diabetes autoantibodies (GADA, IA‐2, ZnT8, and IAA) were enrolled, after excluding 21 subjects with secondary diabetes or refusal to participate. Genetic testing for MODY 1–5 was performed through Athena Diagnostics, and all variants of unknown significance were further analyzed at Exeter, UK. A total of 22 subjects [20 (21%) when excluding two siblings] were found to have a mutation in hepatocyte nuclear factor 4A (n = 4), glucokinase (n = 8), or hepatocyte nuclear factor 1A (n = 10). Of these 22 subjects, 13 had mutations known to be pathogenic and 9 (41%) had novel mutations, predicted to be pathogenic. Only 1 of the 22 subjects had been given the appropriate MODY diagnosis prior to testing. Compared with MODY‐negative subjects, the MODY‐positive subjects had lower hemoglobin A1c level and no diabetic ketoacidosis at onset; however, these characteristics are not specific for MODY. In summary, this study found a high frequency of MODY mutations with the majority of subjects clinically misdiagnosed. Clinicians should have a high index of suspicion for MODY in youth with antibody‐negative diabetes.

Maturity onset diabetes of the young (MODY) is a monogenic form of diabetes caused by a mutation in at least one of the genes known to affect insulin production or secretion. There are at least 13 genes whose mutations have been associated with specific subtypes of MODY. The most commonly occurring MODY subtypes are caused by mutations in glucokinase (*GCK*) (MODY2) and in hepatocyte nuclear factor 1A (*HNF1A*) (MODY3) 1. GCK‐ and HNF1A‐MODY each account for approximately 20–50% of all MODY cases. Approximately 10% of MODY cases are from mutations in hepatocyte nuclear factor 4A (*HNF4A*) (MODY1) and hepatocyte nuclear factor 1B (*HNF1B*) (MODY5). An additional 20% of MODY cases are because of newly discovered or as yet unknown gene mutations for which testing is not yet commercially available 2.

Typically, GCK‐MODY presents clinically as stable, asymptomatic, mild hyperglycemia 3 while HNF1A‐MODY and HNF4A‐MODY present with hyperglycemia and the typical symptoms of diabetes such as polyuria/polydipsia, weight loss, and rarely diabetic ketoacidosis (DKA) 4, 5. In addition to hyperglycemia, patients with HNF1B‐MODY may have renal abnormalities 6, 7. The clinical diagnosis of MODY has been based upon the following criteria: family history of diabetes, insulin independence, and onset by age 25 yr. However, there can be significant clinical overlap of the MODY subtypes with both type 1 and type 2 diabetes mellitus (T1DM and T2DM) 1.

Recently, some studies have attempted to determine clinical and biochemical markers that are specific and sensitive for subtypes of MODY in children. Gandica et al. found *HNF1A* and *GCK* mutations in 5 of 10 (50%) subjects who met a clinical diagnosis of T1DM and at least 2 of the following 3 criteria: negative autoantibodies to at least 1 of either GAD65, IAA, or ICA, hemoglobin A1c level (HbA1c) <7% with an insulin requirement of <0.5 U/kg/day, or at least 3 consecutive generations of family members with diabetes 8. Pihoker et al. evaluated 586 subjects with diabetes, not selected for by referral type or family history, and found that the MODY‐positive group had fewer T2DM features such as lower body mass index (BMI) z‐score, lower prevalence of acanthosis nigricans, and a higher insulin sensitivity, but no significant differences in parental history of diabetes or symptoms at presentation 9. As genetic testing for MODY can be cost prohibitive for many patients, it is necessary to determine which clinical and biochemical characteristics would be more predictive of a positive MODY diagnosis in a given patient 10.

It is important that the diagnosis of MODY be established in affected patients, as there can be significant treatment and hereditary implications depending on the subtype. HNF1A‐MODY and HNF4A‐MODY can be successfully treated with oral sulfonylurea medications as opposed to injected insulin 11, 12, 13, 14, 15 and GCK‐MODY, caused by an altered set point for glucose sensing, may not require any treatment or alterations in diet 16. However, during pregnancy, insulin therapy may be recommended to prevent obstetric complications caused by maternal hyperglycemia 17. Furthermore, clinicians should be aware of the complications associated with the specific subtype of MODY a patient has in order to implement appropriate monitoring and subsequent treatments. Once a mutation is identified in an index case, affected family members should be tested to guide their treatment and clinical monitoring.

The goal of this study was to estimate the frequency of MODY at the Barbara Davis Center (BDC), University of Colorado, among antibody‐negative subjects with diabetes onset less than age 25 and conserved C‐peptide, as well as to assess the clinical characteristics that could distinguish those with genetic mutations for MODY from those without pathologic MODY mutations.

## Methods

### Study population

The BDC is a large, university‐affiliated diabetes center that sees approximately 4000 patients with the diagnosis of T1DM and 180 patients with the diagnosis of T2DM in a year. Subjects actively followed at the BDC who had onset of diabetes before the age of 25 yr, a random C‐peptide ≥0.1 ng/mL, and were negative for all diabetes autoantibodies (GADA, IA‐2, ZnT8, and, if performed within 14 d of onset, IAA) were eligible for this study and offered genetic testing. Patients typically have diabetes autoantibodies measured at diagnosis or with their first visit to the BDC, if they had been diagnosed elsewhere. C‐peptide values were measured for this study, once a subject was determined to have negative autoantibodies. Of the 118 potential participants, 21 were excluded because of iatrogenic diabetes from medications or diabetes secondary to cystic fibrosis, Prader Willi, Fanconi's anemia, other syndromes, or refusal to participate. A total of 97 participants were therefore recruited into the study. At the study visit, subjects and/or parents completed a questionnaire to provide additional information on diabetes, medications, and family history of diabetes or autoimmune disorders. Participants had their height and weight measured, and for all those less than 20 yr old, BMI was calculated and converted to a Z‐score via the Center for Disease Control's (CDC) algorithm. Using Z‐scores for subjects less than 20 yr of age and the raw BMI for subjects over 20 yr of age, the subjects were then classified as ‘underweight’, ‘normal weight’, ‘overweight’, and ‘obese’, as per the CDC's standard guidelines 18. Blood samples were obtained, and a HbA1c was measured by a DCA Vantage Analyzer (Siemans, Munich, Germany). Informed consent and assent when applicable were obtained from each study subject. The Colorado Multiple Institutional Review Board approved all study protocols. A retrospective chart review of the electronic medical record was used to obtain additional clinical data, such as initial HbA1c, initial blood sugar, presence or absence of ketones, DKA and weight loss at diagnosis, and initial pharmacologic treatment.

### Laboratory methods

Autoantibodies to insulin, GAD65, IA‐2, and ZnT8 were measured in the Clinical Immunology Laboratory at the BDC using previously described radio‐immunoassays 19. Random serum C‐peptide (ng/mL) was measured by ELISA immunoassay (ALPCO, Salem, NH, USA) in the BDC Clinical Immunology Laboratory.

Genetic testing for mutations in *HNF1A*, *HNF4A*, *GCK*, pancreatic and duodenal homeobox 1 (*PDX1*), and *HNF1B* was performed for all participants through Athena Diagnostics. DNA sequencing was performed by polymerase chain reaction amplification of purified genomic DNA, followed by Sanger DNA sequencing of the gene's coding region. In addition, at least 10 bases of intronic DNA on either side of each exon containing the highly conserved exon–intron splice junctions were also sequenced. All abnormal sequence variants were confirmed using bi‐directional sequencing. The panel used for this study did not include multiplex ligation‐dependent probe amplification for deletions, which was not available at the beginning of this study.

All variants of unknown significance (VUS) were further analyzed for potential pathogenicity by the Molecular Genetics Laboratory at the Royal Devon & Exeter Hospital, UK. Variants were checked for inclusion in the Human Gene Mutation Database Professional database, dbSNP, the Exome Sequencing Project, the 1000 genomes project, NCBI's PubMed, and the Exeter Laboratory's MODY gene mutation database. Substituted nucleotides and amino acids were checked for conservation across a minimum of five mammalian species, birds, frog, and fish. *In silico* analysis of missense variants was undertaken using the SIFT, PolyPhen2, Align GVGD, and Grantham distance programs (accessed through Alamut v2.3, Interactive Biosoftware, Rouen, France). Synonymous and intronic variants were assessed for potential pathogenic effect on splicing using the splicing prediction programs SpliceSiteFinder‐like, MatEntScan, NNSPLICE, GeneSplicer, and Human Splicing Finder (accessed through Alamut v2.3, Interactive Biosoftware). Variants were classified as benign, likely benign, uncertain, likely pathogenic, or pathogenic based on an assessment of all available evidence obtained, according to practice guidelines for the evaluation of pathogenicity and reporting of sequence variants published by the UK's Association for Clinical Genetic Science 20.

### Statistical analysis

Analyses were performed using Graphpad Prism® 6 and SAS. Statistical comparisons were made between the MODY‐positive and MODY‐negative groups using the Fisher's exact test for categorical variables and the Mann–Whitney test for continuous variables. A two‐tailed p‐value with an alpha level for significance was set at 0.05. In addition, we performed analyses excluding the two related siblings and found similar results (data not shown).

## Results

A total of 20 (21%) subjects, excluding siblings, were found to have pathogenic mutations causative of MODY (MODY‐positive). There were an additional two siblings of MODY‐positive subjects who were also found to be MODY‐positive. Mutations in *HNF1A* (MODY3) were the most commonly found mutations in the MODY‐positive subjects (46%), followed by mutations in *GCK* (MODY 2) at 36%, and mutations in *HNF4A* (MODY1) at 18% (Table 1). None of the subjects tested positive for pathologic mutations in *PDX1* (MODY4) or *HNF1B* (MODY5). Of the 22 total MODY‐positive subjects, 9 (41%) were found to have VUS determined to be likely pathogenic, including one sibling pair. Additionally, one of these nine subjects had two VUS in *GCK* (MODY 2). A total of 4 MODY‐positive subjects (18%) had been clinically diagnosed with MODY at the time of the study, but only 1 of the 22 MODY‐positive subjects had the correct MODY subtype diagnosis (4.5%).

**Table 1:** Pathogenic mutations and their descriptions

Gene	Total subjects with MODY	Total number of VUS‐determined pathogenic	Mutation description (Athena Diagnostics)*	Mutation description (HGVS nomenclature)†
*HNF4A* (MODY1)	4‡	2‡	c.641_648 + 10del	c.575_582 + 10del;p.(?)
			c.998G>A;p.Arg333His‡	c.932G>A;p.(Arg311His)‡
*GCK* (MODY 2)	8	5§	c.431 T>C;p.Leu144Pro	c.431 T>C;p.(Leu144Pro)
			c.460G>C;p.Val154Leu	c.460G>C;p.(Val154Leu)
			c.605 T>C;p.Met202Thr	c.605 T>C;p.(Met202Thr)
			c.1160C>A;p.Ala387Glu	c.1160C>A;p.(Ala387Glu)
			c.1265G>C;p.Arg422Pro	c.1265G>C;p.(Arg422Pro)
*HNF1A* (MODY3)	10‡	2	c.599G>A;p.Arg200Gln	c.599G>A;p.(Arg200Gln)
			c.1136C>A;p.Pro379His	c.1136C>A;p.(Pro379His)
*PDX1* (MODY4)	0	0		
*HNF1B* (MODY5)	0	0		

GCK, glucokinase; HNF1A, hepatocyte nuclear factor 1A; HNF4A, hepatocyte nuclear factor 4A; HNF1B, hepatocyte nuclear factor 1B; PDX1, pancreatic and duodenal homeobox 1; VUS, variance of unknown significance.

* Athena Diagnostic results using reference sequences NM_000457.4 for *HNF4A*, NM_000162.3 for *GCK*, and NM_000545.5 for *HNF1A*.

† Mutations described according to Human Genome Variation Society (HGVS) nomenclature guidelines 21 reference sequences NM_0175914.4 for *HNF4A*, NM_000162.3 for *GC*K, and NM_000545.5 for *HNF1A*.

‡ Including one sibling pair.

§ One subject with two novel mutations.

Table 2 compares the clinical characteristics between MODY‐positive and MODY‐negative subjects at their initial diagnosis of diabetes. At onset of diabetes, the MODY‐positive subjects were significantly older (mean age 13.8 vs. 10.6 yr, respectively, p = 0.008) and had a lower HbA1c [7.9 vs. 10.6% (62.5 vs. 92.2 mmol/mol), respectively, p < 0.0001] compared with the MODY‐negative subjects. MODY‐positive subjects were less likely to present at diagnosis with ketones (14 vs. 60%, respectively, p < 0.0002) or weight loss (9 vs. 59%, respectively, p < 0.0001). None of the MODY‐positive subjects had DKA at diagnosis while 27% of the MODY‐negative subjects presented with DKA (p = 0.053). Most subjects who were MODY‐negative were started on insulin at diagnosis (84%), while the MODY‐positive subjects were more likely to not be started on any pharmacologic treatment at diagnosis (52%).

**Table 2:** Characteristics of MODY‐positive vs. MODY‐negative subjects at onset of diabetes

Characteristics at onset of diabetes	MODY‐negative (N = 75)	MODY‐positive (N = 22)	p‐Value
Female gender	47%	67%	0.0926
Ethnicity			0.16
White	61%	59%	
Hispanic	27%	41%	
Other	12%	0%	
Age, yr	10.6 ± 5.1	13.8 ± 4.4	0.008
HbA1c, % (mmol/mol)	10.6 ± 2.5 (92.2 ± 27.5)	7.9 ± 2.1 (62.5 ± 23.5)	<0.0001
Initial BG, mg/dL	469 ± 262	201 ± 121	<0.0001
Subjects with ketones	60%	14%	0.0002
Subjects with DKA	27%	0%	0.0053
Subjects with weight loss	59%	9%	<0.0001
Pharmacologic treatment			<0.0001
Insulin	84%	43%	
Oral agent	9%	5%	
None	7%	52%	

DKA, diabetic ketoacidosis; HbA1c, hemoglobin A1c level; MODY, maturity onset diabetes of the young.

Characteristics between MODY‐positive and MODY‐negative subjects at study visit are shown in Table 3. Diabetes duration at study visit was similar between the MODY‐positive and MODY‐negative subjects (mean 4.2 vs. 4.0 yr). Both the HbA1c at study visit and the maximum HbA1c recorded, from diagnosis to study visit, were lower in the MODY‐positive group compared with the MODY‐negative group [6.5 vs. 7.7% (48.0 vs. 60.1 mmol/mol), p = 0.004, and 8.4 vs. 10.7% (68.0 vs. 93.2 mmol/mol), p < 0.0001, respectively). Ninety‐five percent of MODY‐positive subjects had a first‐degree relative diagnosed with diabetes compared with 35% of the MODY‐negative subjects (p < 0.0001). At the time of the study visit, the MODY‐positive subjects were significantly more likely to be prescribed only oral agents (27%) or no pharmacologic therapy (36%) compared with the MODY‐negative subjects who were taking insulin in 82% of the cases. The MODY‐positive subjects at the study visit were more likely to have a clinical diagnosis of unspecified diabetes (50%) or MODY (18%), whereas the MODY‐negative groups were mainly diagnosed as T1DM (76%) or T2DM (15%). However, only one of the 22 MODY‐positive subjects had the correct MODY subtype diagnosis (4.5%).

**Table 3:** Characteristics of MODY‐positive vs. MODY‐negative subjects at time of study visit

Characteristics at time of study visit	MODY‐negative (N = 75)	MODY‐positive (N = 22)	p‐Value
Duration since diagnosis, yr	4.0 ± 3.5	4.2 ± 5.8	0.1957
HbA1c, % (mmol/mol)	7.7 ± 1.8 (60.1 ± 20.0)	6.5 ± 0.9 (48.0 ± 10.2)	0.0041
Max HbA1c since Dx, % (mmol/mol)	10.7 ± 2.5 (93.2 ± 27.9)	8.4 ± 2.2 (68.0 ± 23.7)	<0.0001
Family history of diabetes	35%	95%	<0.0001
Weight category			0.76
Underweight	1%	5%	
Normal weight	57%	55%	
Overweight	26%	27%	
Obese	16%	14%	
Pharmacologic treatment			<0.0001
Insulin	82%	37%	
Oral agent	7%	27%	
None	11%	36%	
Diagnosis			<0.0001
T1DM	76%	23%	
T2DM	15%	9%	
Unspecified	9%	50%	
MODY	0%	18%	

HbA1c, hemoglobin A1c level; MODY, maturity onset diabetes of the young; T1DM, type 1 diabetes mellitus; T2DM, type 2 diabetes mellitus.

In total, 27 VUS were reviewed. Nine novel variants were considered to be likely pathogenic mutations, while 17 variants were considered likely benign polymorphisms (Table 4), and one variant (*HNF1A* p.Glu508Lys) was determined as uncertain pathogenicity. As the subject's mother had the same mutation and a diagnosis of diabetes as well, a trial of sulfonylureas while weaning insulin was attempted without success, suggesting that this variant is not pathogenic.

**Table 4:** Variants of unknown significance that were determined likely benign

Gene	Total number of benign variants	Variant description (Athena Diagnostics)*	Variant description (HGVS nomenclature)†
*HNF4A* (MODY1)	7	c.492 + 6G>A	c.426 + 6G>A; p.(=)
		c.493‐4G>A	c.427‐4G>A; p.(=)
		c.504C>G	c.438C>G; p.(=)
		c.505G>A; p.Val169Ile	c.439G>A; p.(Val147Ile)
		c.834G>C; p.Glu278Asp	c.768G>C; p.(Glu256Asp)
		c.1356C>T	c.1290C>T; p.(=)
		c.1387A>G; p.Ile463Val	c.1321A>G; p.(Ile441Val)
*GCK* (MODY2)	1	c.1288C>T	c.1288C>T; p.(=)
*HNF1A* (MODY3)	3‡	c.92G>A; p.Gly31Asp‡	c.92G>A; p.(Gly31Asp)‡
		c.1522G>A; p.Glu508Lys§	c.1522G>A;p.(Glu508Lys)§
		c.1575C>T	c.1575C>T; p.(=)
*PDX1* (MODY4)	5	c.‐18C>T	c.‐18C>T; p.(=)
		c.226G>A; p.Asp76Asn	c.226G>A; p.(Asp76Asn)
		c.725C>T; p.Pro242Leu	c.725C>T; p.(Pro242Leu)
		c.726_728: 3 bp duplication of GCC; codon: 243	c.726_728dup; p.(Pro244dup)
		c.811C>A; p.Pro271Thr	c.811C>A; p.(Pro271Thr)
*HNF1B* (MODY5)	2‡	c.226G>T; p.Gly76Cys	c.226G>T; p.(Gly76Cys)
		c.1207‐18T>C‡	c.1207‐18T>C; p.(=)‡

GCK, glucokinase; HNF1A, hepatocyte nuclear factor 1A; HNF4A, hepatocyte nuclear factor 4A; PDX1, pancreatic and duodenal homeobox 1.

* Athena Diagnostic results using reference sequences NM_000457.4 for *HNF4A*, NM_000162.3 for *GCK*, and NM_000545.5 for *HNF1A*.

† Mutations described according to Human Genome Variation Society (HGVS) nomenclature guidelines 21 reference sequences NM_0175914.4 for *HNF4A*, NM_000162.3 for *GCK*, and NM_000545.5 for *HNF1A*.

‡ Including two unrelated subjects with same mutation.

§ Initially determined as uncertain pathogenicity.

## Discussion

In a large diabetes center, we found a high (21%) incidence of mutations associated with MODY in subjects with negative diabetes antibodies and preserved serum C‐peptide levels at a mean 2.2 yr after diabetes diagnosis. This is a higher incidence than has been described in prior studies which found incidence rates of MODY to be <10% 8, 9. These prior studies only tested for three diabetes autoantibodies (GAD, IAA, and IA‐2); however, this study also tested for ZnT8 antibodies, the presence of which increases the sensitivity of diagnosing T1DM 22, 23. The higher incidence of MODY in this study may be reflective of different inclusion criteria with the necessity of having three to four negative diabetes antibodies and a preserved C‐peptide. Another factor that may have contributed to the higher incidence of MODY in this study is that the population was predominantly white, while the SEARCH population, which found an incidence of 8%, had a higher percentage of minority ethnicities 9. As T2DM is found to occur more often in minority ethnicities than in Caucasians, this study may have included less T2DM subjects 24.

MODY subtype frequencies vary by regions based on screening policies, ethnicity, and age 4, 25, 26. As has been described previously in the UK as well as in the USA, mutations in *HNF1A* were the most commonly occurring MODY subtype, followed by mutations in *GCK* and then by mutations in *HNF4A* 2, 9, 27. No mutations in *HNF1B* or *PDX* were found in this population. Nine subjects had novel mutations considered highly pathologic for MODY, including one sibling pair with the same mutation and one subject having two novel *GCK* mutations. Interestingly, one subject had an *HNF1A* variant (c.1522G>A; p.Glu508Lys) that could not be classified as benign or likely pathogenic. With the subject and family's informed consent and very close monitoring, we attempted to transition the child to an oral sulfonylurea and off of injected insulin; however, 3 months later, while on 0.16 mg/kg/d of glipizide, the subject's average blood glucose was 300 and HbA1c increased to 12.7% (115.3 mmol/mol), necessitating the re‐institution of insulin and suggesting that this variant is not causative of MODY.

While we had 22 subjects who were genetically diagnosed with MODY through this study, only 4 had previously been clinically diagnosed with MODY (18%), and only 1 of these was diagnosed with the correct subtype of MODY (4.5%). Accurate diagnosis is necessary to direct care for the patient, facilitate appropriate diagnosis of relatives, and predict possible future complications and prognosis. Patients with HNF1A‐ and HNF4A‐MODY are sensitive to sulfonylureas and can be transitioned from insulin onto sulfonylureas with improvement in glycemic control and quality of life 11, 12, 14, 28, 29.

MODY‐positive participants were significantly more likely to have a first‐degree relative with diabetes as is consistent with the autosomal dominant inheritance of MODY. We had one subject with negative diabetes antibodies and a preserved C‐peptide who was found to have a mutation in *HNF4A* despite having no family history of diabetes. This likely represents a *de novo* mutation, which has been estimated to occur in approximately 1.2% of referrals for MODY testing 30.

There was no difference between the MODY‐positive and MODY‐negative subjects in regards to gender, ethnicity, or weight. Participants who were MODY‐positive were significantly more likely to be older at diagnosis of diabetes and to have lower HbA1c levels. However, the ranges overlapped for each characteristic, and there was not an absolute age or HbA1c level for which the diagnosis of MODY was excluded. A notable exception however is that none of the subjects with *GCK* mutations had a HbA1c level over 7% (53 mmol/mol), indicating that HbA1c may be used to rule out GCK‐MODY. Similarly, Steele et al. have determined that none of their patients with GCK‐MODY under the age of 40 yr had a HbA1c level over 7.3% 3. At diagnosis of diabetes, none of the MODY‐positive subjects had DKA compared with 27% of MODY‐negative subjects. The frequency of DKA at onset in our MODY‐negative participants is similar to the frequency described in youth with T1DM at diagnosis 31. One subject found to have a mutation in *HNF1A* subsequently went on to develop DKA. As patients with HNF1A‐MODY continue to make endogenous insulin for years, they have a low likelihood of developing DKA; however, this study, in addition to prior published reports of DKA in patients with HNF1A‐MODY, confirms that the presence of a history of DKA does not rule out HNF1A‐MODY 1, 32.

Some of the strengths of this study include a systematic, unbiased method of selecting subjects for genetic testing of MODY, rather than a referral system, and genetic testing for all current clinically available MODY subtypes (MODY 1–5). Limitations of this study include a relatively small number of subjects with MODY, and the fact that random C‐peptide was used instead of fasting or peak C‐peptide from a mixed meal tolerance test. However, random C‐peptide is clinically easier to obtain and has been shown to correlate well with fasting and stimulated C‐peptide 33. In addition, random C‐peptide has been used in several studies to classify types of diabetes in children 34, 35.

In conclusion, in this population of subjects with preserved C‐peptide and negative diabetes autoantibodies, we found a high incidence of MODY with the majority of subjects clinically misdiagnosed. Additionally, a high proportion of genetic mutations were novel. As none of the clinical characteristics alone were sufficient to confirm or rule out MODY, clinicians should have a high index of suspicion for MODY in youth with antibody‐negative diabetes and preserved C‐peptide.
